# Supplementary material for: Rapid turnover of life-cycle-related genes in the brown algae
Source: Genome Biol. 2019 Feb 14;20:35. doi: 10.1186/s13059-019-1630-6 (PMC6374913; doi:10.1186/s13059-019-1630-6)
Supplement: Supplementary file 3 — Protein alignments, corresponding Gblocks html file and CDS sequences for the lineage conserved gametophyte-biased genes. (ZIP 2109 kb) [file 13059_2019_1630_MOESM3_ESM.zip › Con_GA_alignments/Ec-28_000360.aa_ali.fasta-gb.htm]

Ec-28\_000360.aa.fasta.trans.aa\_ali.fasta


## Gblocks 0.91b Results

Processed file: **Ec-28\_000360.aa.fasta.trans.aa\_ali.fasta**  
Number of sequences: **2**  
Alignment assumed to be: **Protein**  
New number of positions: **702** (selected positions are underlined in blue)

```
                         10        20        30        40        50        60
                 =========+=========+=========+=========+=========+=========+
Ec-28_000360     MAMLSCTGPEFFLRSKKLTGKFFLRPRPGIFLQDNSVGSMVSGYRDKAPSTREYPSAGAM
MSTRG.23936.1    ------------------------------------------------------------
                                                                             


                         70        80        90       100       110       120
                 =========+=========+=========+=========+=========+=========+
Ec-28_000360     AGTAVVVEKERRIRLEASNRRRAHILRAKLNEEAEEEKYRLALSKRQHEQQEYARSVRKN
MSTRG.23936.1    ------------------------------------------------------------
                                                                             


                        130       140       150       160       170       180
                 =========+=========+=========+=========+=========+=========+
Ec-28_000360     IRSWRKQGSPAPVGDSTVQKPAQPWEQKNRGGTPVISGTGQSATRPAKLTSTRKGNANVN
MSTRG.23936.1    ------------------------------------------------------------
                                                                             


                        190       200       210       220       230       240
                 =========+=========+=========+=========+=========+=========+
Ec-28_000360     WSPSNSVGGVCALEEWEDVAEMTGISAWTRHEAAGRGETISPKLTAVIDRSYLVSSDVGV
MSTRG.23936.1    ------------------------------------------------------------
                                                                             


                        250       260       270       280       290       300
                 =========+=========+=========+=========+=========+=========+
Ec-28_000360     EGGDVAGWDSREQSRGGRWANLPPELRAIKDRWLGSSSEEITPQAATSDITVSAGATITA
MSTRG.23936.1    ------------------------------------------------------------
                                                                             


                        310       320       330       340       350       360
                 =========+=========+=========+=========+=========+=========+
Ec-28_000360     PAHGGWQDQHSYFQEYRSRVVPRDKDFRYAHPNILVEGSAPHDNHGRLEGAETKTTSEMV
MSTRG.23936.1    ------------------------------------------------------------
                                                                             


                        370       380       390       400       410       420
                 =========+=========+=========+=========+=========+=========+
Ec-28_000360     DEMEKQGHGCGEDSNSTIRRISSDSLECSRLVENGLQVTRSAEGRDAHHPRLRQADAESL
MSTRG.23936.1    ------------------------------------------------------------
                                                                             


                        430       440       450       460       470       480
                 =========+=========+=========+=========+=========+=========+
Ec-28_000360     KDSPQHQHQQEGLLHPTANDNELLGKSSRDDVTPRGSGEPGDRRPPDTQEGKVSGLAEDA
MSTRG.23936.1    ------------------------------------------------------------
                                                                             


                        490       500       510       520       530       540
                 =========+=========+=========+=========+=========+=========+
Ec-28_000360     AAPSTEEWFGDILPLQRSEVVGDPRDGHRRAKVKNQLQDLRNRLPNGATGEVPTAVSPKP
MSTRG.23936.1    ------------------------------------------------------------
                                                                             


                        550       560       570       580       590       600
                 =========+=========+=========+=========+=========+=========+
Ec-28_000360     VNVNDALLPAQQATSFASARTQQPWKGGAQPGVGEAEEVGDHGRRRDIDETLGDNKGGAQ
MSTRG.23936.1    ------------------------------------------------------------
                                                                             


                        610       620       630       640       650       660
                 =========+=========+=========+=========+=========+=========+
Ec-28_000360     DTLRPATLVQKVPSHGGEGTVNQRVDGTLTLDIAPLEERERESTGEQQGAQETLITRSDV
MSTRG.23936.1    ----------------------------XFFHPAPLS-----------------------
                                                                             


                        670       680       690       700       710       720
                 =========+=========+=========+=========+=========+=========+
Ec-28_000360     ADVEGRSGQGNN-YDTTNTRSRPSELFDSLDVSARASQDGGGLLTRAGGRSDEIGAPAIP
MSTRG.23936.1    GVVEGRPGNGENGSDTTSTTSRPSELIDSLDLSVSAPPYS-DTRARDRGRSGSTVVAAIP
                   ##########  ######################         ###############


                        730       740       750       760       770       780
                 =========+=========+=========+=========+=========+=========+
Ec-28_000360     ATARFAPGTEVITPPKVRSPDKGPENTKIRNRDHVLNDQNFSTMAALTVETHSGRFTATS
MSTRG.23936.1    ASVQSAFTAGTVSCPTPQGLGE-KEGRNVRSLNITIDNPTVVVAVASTTRINS-RTLAGV
                 ###############                                       ######


                        790       800       810       820       830       840
                 =========+=========+=========+=========+=========+=========+
Ec-28_000360     GDAPPPRRDRPLVKVVPEETTEPIT--RRLPNHATERVAILRPERAVQSPPTVPSRKLTT
MSTRG.23936.1    ASLSPPREETPDKTVPAETTAVPDVGGRTLHALTGTAAKISGPERHVGGSPKTLPKTTPS
                 #######################                ############         


                        850       860       870       880       890       900
                 =========+=========+=========+=========+=========+=========+
Ec-28_000360     KPTGDGKCKLREPPHTSRLRAPGWGREAVITAILPAKVTPGMAKDGSSPATTASHNGVAP
MSTRG.23936.1    KTSGASKTAKKQAPGVSRLRAPGWGREASISSIPPATNVVGVAKLGTVPAKPARDVGATP
                 ############################################################


                        910       920       930       940       950       960
                 =========+=========+=========+=========+=========+=========+
Ec-28_000360     TDPAVLPWDLLTPVTEAATVDERSVISHGTHGTTRMTPASSVTSGCRPPQGEASGAIQGR
MSTRG.23936.1    SATAPLPWDLLTPVTEAPTADERSVASHGTRGTTRITPASSVASGCRKDEGGASGALRER
                 ############################################################


                        970       980       990      1000      1010      1020
                 =========+=========+=========+=========+=========+=========+
Ec-28_000360     VSRRYPHRLQADANRYAVAAAPVVCALESPAETSGFGDGRNQNGSEKRVVWGSGTVASGA
MSTRG.23936.1    VSHRSPHCLQPGVLHRATP-APGICKKKAHSRGKEFADGRSRIYRENRAMLGTSPIDSGA
                 #################                  #########################


                       1030      1040      1050      1060      1070      1080
                 =========+=========+=========+=========+=========+=========+
Ec-28_000360     HSAPGTTSARSRRRDAPAGGALTSSSAYRASPLSSTADRVGTGGSVFSSASGETNLGRQF
MSTRG.23936.1    RSAQCQRSVPWTGPTSPRGGPLSSVSAYRASPSCGTSEGVRTRCSTFSSASEERTPGRQA
                 ############################################################


                       1090      1100      1110      1120      1130      1140
                 =========+=========+=========+=========+=========+=========+
Ec-28_000360     GGGYVAAMGRRARSMARCEAARR-LAAAMAADRGRPSTPISERARWGKGKES--QEEAME
MSTRG.23936.1    GGGYIAAMGRRARSVARREAARRSAVAAMVAHKTRPSTPISERARWGKGKESQVEIERKK
                 #######################   ##########################    ####


                       1150      1160      1170      1180      1190      1200
                 =========+=========+=========+=========+=========+=========+
Ec-28_000360     LARRKCAAANTSTMLPADPRHVRAG----ATSASSTAFQKDSTPYDADTSMGGCQGKVPS
MSTRG.23936.1    LLRRRAEYAEELQRKAKEDAKAVAERRAVATTTETTMLPADPRHVRAGAGSGACFTPQHG
                 #########                    ###############################


                       1210      1220      1230      1240      1250      1260
                 =========+=========+=========+=========+=========+=========+
Ec-28_000360     AIEANFTHDTTRPEAIDS----RHKDKPPCLAPGSKANSPVSSRRCDGLRVGVGGGDGG-
MSTRG.23936.1    AVTPTSTEAGTIREAACFGSFPYRGNKAPHLLLDSLVESPAFCRQDHSGRQSSAGRTKGD
                 ###############           ################################# 


                       1270      1280      1290      1300      1310      1320
                 =========+=========+=========+=========+=========+=========+
Ec-28_000360     ----LHRRQKAKRQIAKSTTQEEEEERLTASIARLDTLLREGDGEVSGRASDAPMPGGSG
MSTRG.23936.1    DSIAFVGQELGTRAEPRGGSPDGEEERLMASIARLDTLLREDNG-----KPHAPTAAVN-
                                        #####################                


                       1330      1340      1350      1360      1370      1380
                 =========+=========+=========+=========+=========+=========+
Ec-28_000360     GVTPSSKSGGRVERRPVRAPVGGIAKTRVRRGATKEIT-SSAGPVVNVEPKAGLVSVAAA
MSTRG.23936.1    --SVSRKDSGGMECKAARMQAGGVAKARVRRGGRETKAYGSAGSVPVVAPKTPLT-QSVP
                     ############################        ##############      


                       1390      1400      1410      1420      1430      1440
                 =========+=========+=========+=========+=========+=========+
Ec-28_000360     VPVHTTAVVDAGAAIMASPGLFPPIFGRVPAHTGGGREIGRYEPPDAVQQTSNGPSRGVA
MSTRG.23936.1    VPTATSLATNAVVPAQISPGMFPPLFGRTPAGPGVQPPRARCLRPGEHEMDQTGPPHKML
                 ############################################################


                       1450      1460      1470      1480      1490      1500
                 =========+=========+=========+=========+=========+=========+
Ec-28_000360     YREPASHCPIGRAASPLSYRYCADDEQRRAKRADGGPVDAAPGHYCQ-EGRRPAGAASRW
MSTRG.23936.1    HETPASHVPVEMAASPLSNRMRVGEERRRVERSDGGFTDAPYDNFQQRQAGQLAWETNRS
                 ###############################################      #######


                       1510      1520      1530      1540      1550      1560
                 =========+=========+=========+=========+=========+=========+
Ec-28_000360     VGDGND----HYDARAPPRYPDQREELRVPQTPCSPARYRGVDNSYDNHRGWHDHSRGGS
MSTRG.23936.1    IGYTNDRNDYDYRASVQPRYFVRREGEMFPPGTRTSARYWDPAHGDDRPEGHYVYGRGSG
                 ######     #################################################


                       1570      1580      1590      1600      1610      1620
                 =========+=========+=========+=========+=========+=========+
Ec-28_000360     YYRRDDLRAAIDPRDAPGVVDHARWIGESRPEEYGDDRGGETYWIRRDGGTTFQHEDRKG
MSTRG.23936.1    ASGR-------FRRDDASIIPP---------QR---------------------------
                 ####                                                        


                 
                 ==
Ec-28_000360     HF
MSTRG.23936.1    -Y
```

```
Parameters used
Minimum Number Of Sequences For A Conserved Position: 2
Minimum Number Of Sequences For A Flanking Position: 2
Maximum Number Of Contiguous Nonconserved Positions: 8
Minimum Length Of A Block: 10
Allowed Gap Positions: None
Use Similarity Matrices: Yes
```

```
Flank positions of the 17 selected block(s)
Flanks: [663  672]  [675  696]  [706  735]  [775  803]  [820  831]  [841  977]  [996  1103]  [1107  1132]  [1137  1149]  [1170  1215]  [1227  1259]  [1284  1304]  [1325  1352]  [1361  1374]  [1381  1487]  [1494  1506]  [1512  1564]  

New number of positions in Ec-28_000360.aa.fasta.trans.aa_ali.fasta-gb:  702  (43% of the original 1622 positions)
```
